# The effect of social care nurses on health related quality of life in patients with advanced cancer: A non-randomized, multicenter, controlled trial

**DOI:** 10.1007/s11136-024-03780-3

**Published:** 2024-09-13

**Authors:** Daniel Schindel, Johann Frick, Pimrapat Gebert, Ulrike Grittner, Anne Letsch, Liane Schenk

**Affiliations:** 1 Institute of Medical Sociology and Rehabilitation Science, Charité – Universitätsmedizin Berlin, Corporate Member of Freie Universität Berlin, Humboldt-Universität zu Berlin, Charitéplatz 1, 10117 Berlin, Germany; 2 Institute of Biometry and Clinical Epidemiology, Charité – Universitätsmedizin Berlin, Corporate Member of Freie Universität Berlin, Humboldt-Universität zu Berlin, Charitéplatz 1, 10117 Berlin, Germany; 3 Berlin Institute of Health at Charité – Universitätsmedizin Berlin, Anna-Louisa-Karsch-Str. 2, 10178 Berlin, Germany; 4 Department of Medicine II, Hematology and Oncology, University Hospital Schleswig-Holstein, Arnold- Heller-Straße 3, 24105 Kiel, Germany; 5 Charité Comprehensive Cancer Center, Charité – Universitätsmedizin Berlin, Corporate Member of Freie Universität Berlin, Humboldt-Universität zu Berlin, Berlin Institute of Health, Charitéplatz 1, 10117 Berlin, Germany

## Introduction

Patients with advanced cancer and a limited prognosis suffer from particular physical, psychological, and social burden [1, 2]. Identifying and appropriately addressing patients’ complex care needs is a tremendous challenge [3]. Overtreatment and redundant aggressive therapies at the end of life have been discussed as well as to positive therapy expectations on the part of the patients [4, 5]. Patients’ unexpressed needs are associated with higher psychosocial distress and decreased quality of life (QoL) [6]. Targeted support services may address patients’ symptom awareness, health literacy or patient-physician communication and thereby increase the likelihood of patients expressing personal care needs [7]. Care needs associated with the disease are broadly defined and include supportive conversations about anxieties and concerns in relation to cancer recurrence, the further course of the disease, confrontation with death, physical changes, treatment side effects, sexuality, partnership and family problems, workplace problems, and financial security [7]. Thus, psycho-oncological and supportive care interventions are an important complement to medical treatments that can improve patients’ quality of life [8, 9]. QoL is a multidimensional concept that includes physical, emotional, social, and functional well-being [10]. It is crucial to include the patient’s voice on their QoL not only in clinical decision-making but also in the development of therapies and regulatory decision-making [11, 12]. As curatively-treatments in advanced stages of cancer are no longer indicated, improving quality of life becomes a central therapeutic goal [13].

Previous psycho-oncological and supportive care offerings focusing on QoL encompassed a variety of forms, often combined with navigational support. Patient navigation involve roles and activities designed to help cancer patients overcome healthcare barriers and access high-quality health and psychosocial care. They provide support to organize tailored assistance to meet the specific needs of patients and their caregivers, addressing identified obstacles and personalized care objectives to ensure the timely and optimal use of available services within the cancer care continuum [14, 15]. Interventions range from continuous contact persons [16, 17] and organized (online) peer support [18, 19], to counselling cross-sectoral nurse navigators who aim to coordinate care between ambulatory and inpatient care) [20, 21] to supportive care at home [17] and nurse-led outpatient “enhanced supportive care” [22]. Due to their early contact with patients and their pre-existing qualifications, inpatient nursing staff seem to be well suited to closely monitor the health situation of patients by means of standardized assessment tools and to offer needs-oriented support services [20, 23–26]. Changes in quality of life could thus be timely identified and serve as an indicator of supportive care needs that require further clinical attention [27].

A recent umbrella review illustrates broad evidence on the effectiveness of interventions based on navigational support. On the one hand, there was a large number of potential target areas (psychosocial support, care coordination, treatment knowledge, decision making, e.g.), and on the other hand, there was clear evidence of the effectiveness of interventions in the areas of adherence to surveillance, decision making and treatment knowledge, patient satisfaction and quality of life [14, 28, 29]. However, further research is required to develop appropriate supportive care models that meet the multidimensional care needs of cancer patients and their families [30], while taking into account the barriers and facilitators related to the implementation of navigation support, e.g. low health literacy or lack of referral from providers [14].

The aim of this study was to examine if advanced cancer patients with poor prognosis benefit from navigational support and coordinating psychosocial counselling services through social care nurses (SCN), particularly with respect to improvements in health-related quality of life over the study period of one year. Further, we analyzed the impact of SCNs on patients’ health literacy, treatment participation, decisional conflicts, and coherence.

## Methods

This study was conducted as prospective, non-randomized, multicenter, and controlled study over a 12-month observation period [31, 32]. Between February 2018 and February 2020, participants were recruited from the hematology and oncology departments and outpatient clinics of four hospitals in three German cities.

### Study procedure

All patients in the participating hospitals received standard care according to international medical guidelines. In addition, patients in the intervention group received additional services from their personal social care nurse (SCN). They assessed patient needs at least monthly in order to systematically identify gaps in patient care. The roles, functions and background of the SCNs appointed are described following Kelly et al. [15]. The six SCNs were employed by the four participating hospitals. They worked in regular shifts on the oncological wards with a fixed quota of hours off for additional navigational and counselling tasks in the study. Care was provided beyond the patients’ hospital stays and was carried out by the same SCN as far as possible. A key function of the SCNs was the coordination of medical, palliative and psychosocial support offers. Additional functions were to educate and counsel patients about the health care system (e.g., application for health and social legal services) and navigate services (e.g., contact to support groups) to reduce barriers to receiving timely services. SCNs have a professional background. The prerequisite for further training as a SCN was nursing training, or a degree in social pedagogy or social work. The majority of the six SCNs had additional training in psycho-oncology. The SCNs were approached by senior clinicians. As the activity involved an additional professional qualification, but also greater autonomy in daily work, it was perceived as a gratification.

Assignment to the two study groups was based on patients’ statutory health insurance. Patients with membership in a company health insurance funds were enrolled in the intervention group. Patients of any other statutory health insurance type could take part in the control group in this trial. Background for the non-randomization is the funding framework. Statutory health insurances receive funding for testing new innovative forms of care, which they offer to their own insureds. Due to the specific inclusion criteria, the number of potential patients among the insured persons of the participating health insurers was limited. Randomization within this group was not feasible, thus an external comparison group recruited out of all other statutory insurance companies was elected.

### Participants

By inclusion criteria the study focused on patients with advanced oncological diseases and poor prognosis. Patients with at least one of the 16 predefined cancer diagnoses combined with specific therapies and surgeries were enrolled (see online supplementary document, Table S1) [31]. The minimum age for participation in the study was 18 years. Advanced dementia or severe addiction disorders were exclusion criteria [31].

#### Intervention group (IG)

Patients eligible for the intervention were recruited by the SCNs. All patients were informed and consulted about the study through detailed information and paper study documents. The SCN aimed to recruit patients as soon as possible after diagnosis and before surgery or treatment. After enrollment, they were actively contacted and interviewed by their personal SCN at least once a month over one year by telephone or face-to-face meetings in the clinic. Patients in the intervention group completed monthly the EORTC QLQ-C30 and they did not receive any monetary compensation.

#### Control group (CG)

Patients eligible for the control group were recruited by regular study nurses. They received standard care based on treatment according to evidence-based cancer guidelines. Patients in the control group did not receive any additional care or services and had no contact with the SCNs. They received at least a monetary compensation of EUR 20.00 for each survey participation.

### Study outcomes and questionnaires

**Fig. 1:**
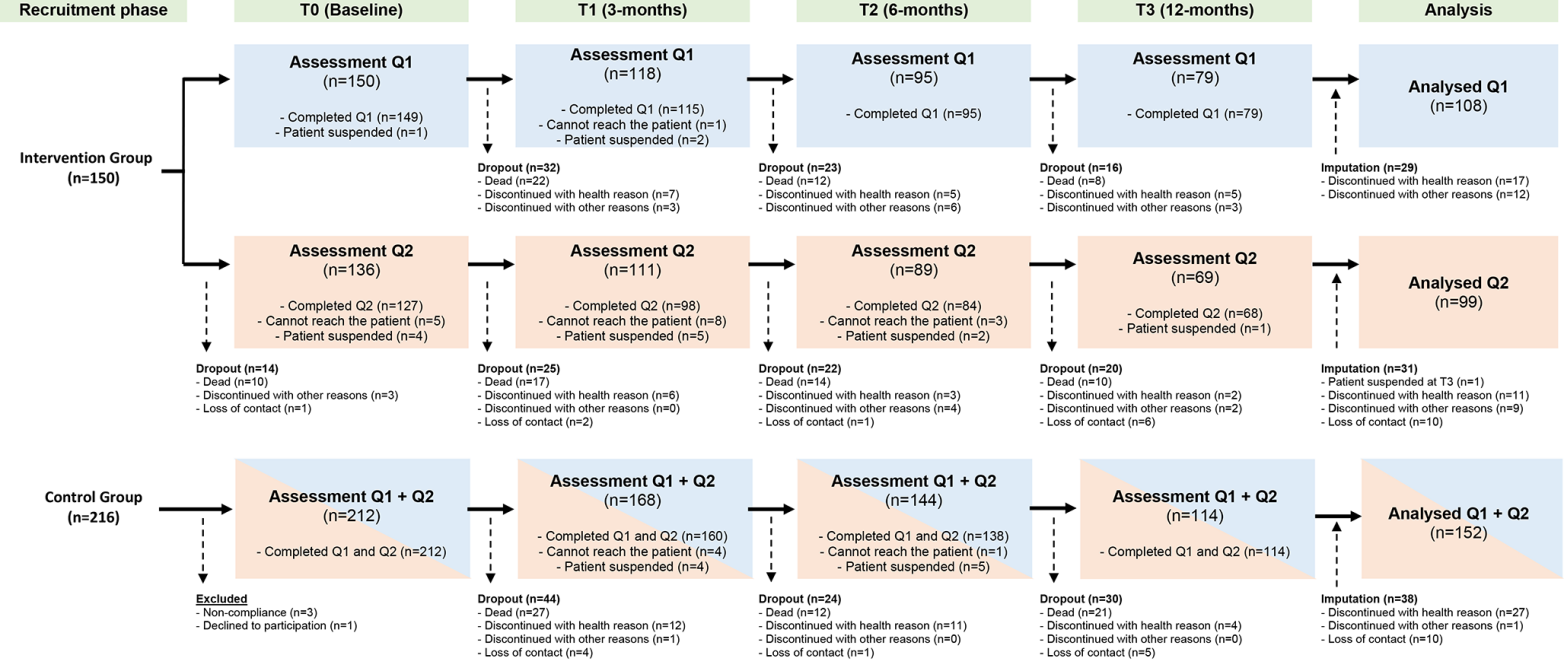
CONSORT flow diagram. Q1 (blue boxes) = Questionnaire 1 assessing quality of life (EORTC QLQ-C30 Questionnaire). Q2 (orange boxes) = Questionnaire 2 assessing demographic data, health care utilization, health literacy, patient-physician communication, participation preference, illness coherence, decision conflict questionnaires. Abbreviations: EORTC, European Organisation for Research and Treatment of Cancer Quality of Life Questionnaire Core 30; QoL, Quality of Life

The primary outcome, Quality of Life (QoL), was the average of two items (global health status and quality of life (GHS/QoL)) selected from the validated EORTC QLQ-C30 questionnaire (European Organisation for Research and Treatment of Cancer quality of life 30-item questionnaire) [10, 33, 34]. It is a cancer specific, modular quality of life questionnaire including five functional scales (physical, role, cognitive, emotional, and social), three symptom scales (fatigue, pain, and nausea/vomiting), and a global scale for health and quality of life. The remaining individual items capture additional symptoms frequently reported by cancer patients (dyspnea, appetite loss, insomnia, constipation, and diarrhea), as well as the perceived financial impact of the disease and treatment. After a linear transformation, the standardized score ranges between 0 and 100. For GHS/QoL and functional scales, a high score represents a good GHS/QoL or functioning. Conversely, a high score expresses strong symptoms for symptom scales [33]. In our study the EORTC QLQ-C30 questionnaire (see Fig. 1, questionnaire 1 (Q1)) was collected monthly in the intervention group by a social care nurse. In the control group a regular study nurse collected the EORTC QLQ-C30 at baseline, after 3, 6, and 12 months (see Fig. 1).

Secondary outcomes were subscales for symptoms and functioning of the EORTC QLQ-C30, and six patient-reported outcomes measures (PROMs). We surveyed a 6-item questionnaire from the European Health Literacy Survey to assess the intervention’s impact on patients’ health literacy (HLS-EU-Q6 [35]),. Each of the six questions offers five response options. An average score is determined and classified into one of three categories: insufficient health literacy [range 1–2], problematic health literacy (2–3), and sufficient health literacy [3–4]. Further, we used the Patient Reaction Assessment (PRA-D [36]), a self-report tool designed to assess the quality of the patient-physician relationship as perceived by the patient. In this study, we modified five communication-related questions to use a five-point Likert scale. A total score was calculated and rescaled, whereas higher scores indicate better doctor-patient communication. We have assessed patients’ preferences for involvement and information in medical decision-making using two item sets of the German modified version of the Autonomy Preference Index (API-Dm [37]),. The information preference section includes seven items, yielding scores between 0 and 28. The participation preference section comprises four items, with scores ranging from 0 to 16. Each patient’s total preference score is then converted to a scale from 0 to 100, with higher scores indicating a greater preference for information or participation. Further we surveyed the Decisional Conflict Scale (DCS-10 [38]), a self-report questionnaire designed to evaluate decision conflicts among patients. The short version includes ten items divided into four subscales: uncertainty, information, values clarity, and decision support. Each item is rated on a three-point Likert scale (0 = “yes”, 2 = “unsure”, 4 = “no”). The total score was rescaled to range from 0 (no decision conflicts) to 100 (high decision conflicts). To assess individuals’ beliefs and feelings about their illness we have surveyed a part of the Illness Perception Questionnaire (IPQ [39]),. The full instrument comprises 64 items across 8 scales. For this study, the illness coherence subscale, which includes five items, was utilized in the evaluation questionnaire. The scores from the items were totaled and then averaged. Higher the values, the more confusing and puzzling the symptoms are for the patient. The following sociodemographic data were collected and considered for analysis: age, gender, family status, level of care, educational status, professional background, social support through close people, and neighbors (assessed using the Oslo Social Support Scale (OSSS-3 [40]), ), and subjectively reported social status through the MacArthur Scale [31]. Data for the secondary outcomes and sociodemographic characteristics were collected by trained study nurses using a questionnaire in face-to-face, postal or telephone interviews at baseline, after 3, 6, and 12 months in both groups (see Fig. 1, questionnaire 2 (Q2)).

### Sample size and statistical analysis

#### Sample size calculation

A sample size calculation was performed to detect a difference of global health status/QoL in the EORTC QLQ-C30 with a Cohen’s d effect size of 0.4 [33, 41]. A sample size of 100 patients in the intervention group and 150 patients in the control group was required, yielding a power of 80% at a two-sided significance level of 5% using a two-sample t-test. To account for a high dropout rate due to high severity of the cancer entities included and due to the lack of participation benefit for the control group we originally aimed to include 150 patients in the intervention and 200 patients in the control group (350 in total). Recruitment was further expanded during the course of the study due to the high post-operative death rate (hence, the recruitment was done before surgery or therapy).

#### Statistical analysis

Descriptive summary statistics such as mean and standard deviation (SD) are presented for continuous variables and absolute and relative frequencies for categorical variables. The inverse probability of treatment weighting (IPTW) method was applied to account for selection and imbalances in baseline characteristics between the intervention and control group in relation to socio-demographic data (e.g. age, education) and different measures at baseline (e.g. GHS/QoL; PRA-D) in this non-randomized study [41, 42]. A discussion of the unbalanced baseline characteristics before applying IPTW has been published elsewhere [32]. To deal with missing data, we performed multiple imputations with chained equations (MICE) for all data that were assumed to be missing at random (MAR). Missing values caused by premature death were not imputed for the primary analysis but in the sensitivity analyses (please see more detail in the online supplementary, Table S3). Changes in GHS/QoL measured by the EORTC QLQ-C30 were estimated using an IPTW-adjusted linear mixed model with a random intercept for the patients and the study group (intervention/ control) as a fixed factor. The model was adjusted for baseline scores, study site, and follow-up times (3, 6, and 12 months) as covariates. Other potential confounders (e.g. education, diagnosis, marital status etc.) were used in estimating the weighting score, therefore they were not included into the final regression model. Interactions between group allocation and time points were explored. Adjustment for multiple tests was made only for the primary outcome that was measured at three different time points using the Benjamini-Hochberg method [43] for controlling the false discovery rate (FDR) of 0.05.

We also conducted exploratory analyses to examine potential changes in secondary outcomes using the same statistical method as above. A sensitivity analysis was performed to assess the robustness of the conclusions: for this purpose, pattern-mixture models [44, 45], joint models [46], and worst-case scenario were utilized to identify plausibly missing data mechanisms as for example missing not at random (MNAR) or MAR. Our results will be seen as robust if none of the sensitivity analyses’ results differs from the primary analysis. All statistical tests were performed using Stata IC15 (StataCorp, 2017, College Station, TX, USA). Further details of the IPTW, missing imputation and sensitivity analysis are provided in the online supplementary document (pages 2 and 3).

## Results

### Patients’ baseline characteristics and dropout rates

Of the 366 patients recruited for the study, 362 could be included in the analyses (intervention group (IG) = 150 and control group (CG) = 212). The mean patient age in the intervention group was 66 years (SD = 13) and 62 years in the control group (SD = 13). 58.0% (IG) and 62.3% (CG) of patients were male (Table 1). The most common diagnosis in the control group was acute leukemia (23.6%), while in the intervention group, malignant neoplasms of bronchus and lung (22.0%) and metastatic colorectal cancer (22.7%) had the highest shares. Patients’ characteristics differed between study groups at baseline in terms of age, education, diagnosis, and patient reported outcome measures (Table 1). However, after IPTW, the distribution of baseline characteristics among groups was similar (Online supplementary document Figure S1).

**Table 1:** Baseline characteristics after imputation (*n* = 362)

Baseline characteristics	Number of missing data	Total** (*n* = 362)	Before IPTW			After IPTW		
			Control group (*n* = 212)	Intervention group (*n* = 150)	SMD	Control group (*n* = 212)	Intervention group (*n* = 150)	SMD
**Age (years)** – Mean (SD)	0	63 (13)	62 (13)	66 (13)	0.31	63 (12)	62 (14)	-0.07
Sex*	0				0.09			0.07
Male		219 (60.5%)	62.3%	58.0%		61.3%	58.0%	
Female		143 (39.5%)	37.7%	42.0%		38.7%	42.0%	
**Family status**	29 (8%)				-0.18			-0.01
Married		226 (67.9%)	65.8%	71.9%		69.3%	68.9%	
Single		44 (13.2%)	12.3%	13.8%		11.9%	13.1%	
Divorced/Widowed		63 (18.9%)	21.9%	14.3%		18.8%	18.0%	
**Education**	26 (7%)				-0.36			< 0.001
Low		29 (8.6%)	4.8%	15.4%		8.6%	8.6%	
Medium		101 (30.1%)	30.0%	32.6%		30.5%	30.3%	
High		206 (61.3%)	65.2%	52.0%		60.9%	61.2%	
**Social support (Osss-3)** – Mean (SD) Missing = 30	30 (8%)	11.0 (2.1)	11.1 (2.0)	10.8 (2.1)	-0.14	10.9 (2.0)	10.8 (2.0)	-0.01
**Time since diagnosis (months)** – Median (IQR)	0	6 (2, 22)	7 (2, 19)	5 (1, 26)	0.12	7 (2, 20)	4 (1, 23)	-0.01
**Diagnosis (ICD-10-Codes)**	0				0.25			-0.03
Acute leukemia		69 (19.1%)	23.6%	12.7%		20.1%	23.2%	
Aggressive lymphoma		58 (16.0%)	17.9%	13.3%		15.9%	14.9%	
Malignant neoplasm of bronchus and lung		62 (17.1%)	13.7%	22.0%		18.0%	16.1%	
Metastatic colorectal cancer/colon carcinoma		78 (21.6%)	20.7%	22.7%		21.8%	22.5%	
Malignant neoplasm of pancreas		32 (8.8%)	7.1%	11.3%		7.7%	8.1%	
Multiple myeloma and malignant plasma cell neoplasms		24 (6.6%)	8.5%	4.0%		6.6%	4.9%	
Metastasized malignant neoplasm of breast		9 (2.5%)	2.4%	2.7%		2.9%	2.4%	
Others		30 (8.3%)	6.1%	11.3%		7.0%	7.9%	
**Primary and secondary outcomes**								
**EORTC QLQ-C30** – Mean (SD)								
Global health status/QoL (Primary Endpoint)	7	50.6 (21.9)	52.4 (21.7)	47.6 (21.8)	-0.22	50.7 (21.2)	50.1 (21.6)	-0.02
**EORTC QLQ-C30 functional and symptom subscales** – Mean (SD)								
Physical functioning	4 (1%)	57.6 (25.4)	60.1 (25.8)	53.9 (24.2)	-0.25	57.5 (26.0)	57.6 (24.9)	0.01
Emotional functioning	5 (1%)	57.2 (27.1)	58.6 (27.5)	54.9 (26.2)	-0.14	56.5 (26.9)	56.4 (25.2)	-0.01
Pain	1 (< 1%)	37.3 (36.1)	32.5 (34.9)	44.1 (36.7)	0.33	38.0 (36.5)	36.3 (35.1)	-0.05
Diarrhoea	1 (< 1%)	21.9 (32.7)	24.5 (33.6)	18.3 (31.0)	-0.19	21.9 (31.9)	20.6 (32.2)	-0.04
Financial difficulties	5 (1%)	17.9 (30.4)	22.2 (33.8)	12.0 (23.5)	-0.35	18.4 (31.0)	17.0 (27.3)	-0.05
**PRA-D -** Mean (SD)	27 (7%)	29.8 (6.5)	30.6 (6.0)	28.3 (6.9)	-0.36	29.4 (7.3)	29.7 (6.4)	0.04
**API-DM -** Mean (SD)								
Preference for participation	26 (7%)	52.8 (14.9)	53.7 (15.2)	51.2 (14.0)	-0.17	52.8 (14.8)	51.3 (14.0)	-0.10
Preference for information	25 (7%)	96.1 (6.5)	96.5 (6.0)	95.6 (7.2)	-0.15	96.5 (6.4)	96.0 (6.1)	-0.07
**IPQ-R**: Illness coherence – Mean (SD)	30 (8%)	16.2 (4.3)	16.6 (4.4)	15.5 (3.9)	-0.25	16.3 (4.3)	16.3 (4.1)	-0.01
**DCS** – Median (IQR)	27 (7%)	15 (0, 40)	15 (0, 35)	20 (5, 45)	0.17	15 (0, 35)	15 (0, 35)	0.01
**HLS-EU-Q6** – Mean (SD)	119 (33%)	2.8 (0.7)	2.80 (0.63)	2.67 (0.61)	-0.21	2.73 (0.66)	2.76 (0.61)	0.06

*Sex was not included in the IPTW. **Total is presented before imputation

The baseline characteristics before IPTW are discussed elsewhere in detail [32]

Abbreviations: API-DM: German modified Version of the Autonomy Preference Index; DCS: Decision Conflict Scale; EORTC QLQ-C30: European Organisation for Research and Treatment of Cancer Quality of Life Questionnaire Core 30; HLS-EU-Q6: Health Literacy Questionnaire; ICD: International Classification of Diseases; IPQ-R: Illness-Perception-Questionnaire; IPTW: Inverse probability of treatment weighting; IQR: Interquartile range (25th percentile: 75th percentile); Osss-3: Oslo social support scale; PRA-D: German Version of the Patient Reactions Assessment; QoL: Quality of Life; SD: Standard deviation; SMD: Standardized mean difference

Median follow-up time in the intervention group was 316 days (Interquartile range (IQR): 126, 339 days) and 356 days in the control group (IQR: 172, 366 days). Dropout rates over time and the reasons for these are presented in the CONSORT flow diagram (Fig. 1). Cumulative attrition in the two groups was 14.4% at 3 months, 31.5% at 6 months, and 46.8% at 12 months [44]. The overall attrition rate did not differ between the study groups (IG: 47.3% (*n* = 71/150); CG: 46.2% (*n* = 98/212)) and the majority of attritions were due to premature death (IG: 59.2% (42/71); CG: 61.2% (60/98)) [47]. Screening data for the intervention group were not available due to data protection in relation to the small number of patients insured in the defined health insurances. In total, 616 patients were screened in the control group. The most frequent reasons for non-participation were lack of interest in participating (*n* = 120), health status (*n* = 82), discharge from hospital (*n* = 55), health insurance suitable for intervention group (*n* = 54) and other reasons (*n* = 53; e.g., age < 18, dementia, addiction disease).

### Change of quality of life between groups over the study period of one year

#### GHS/QoL

**Fig. 2:**
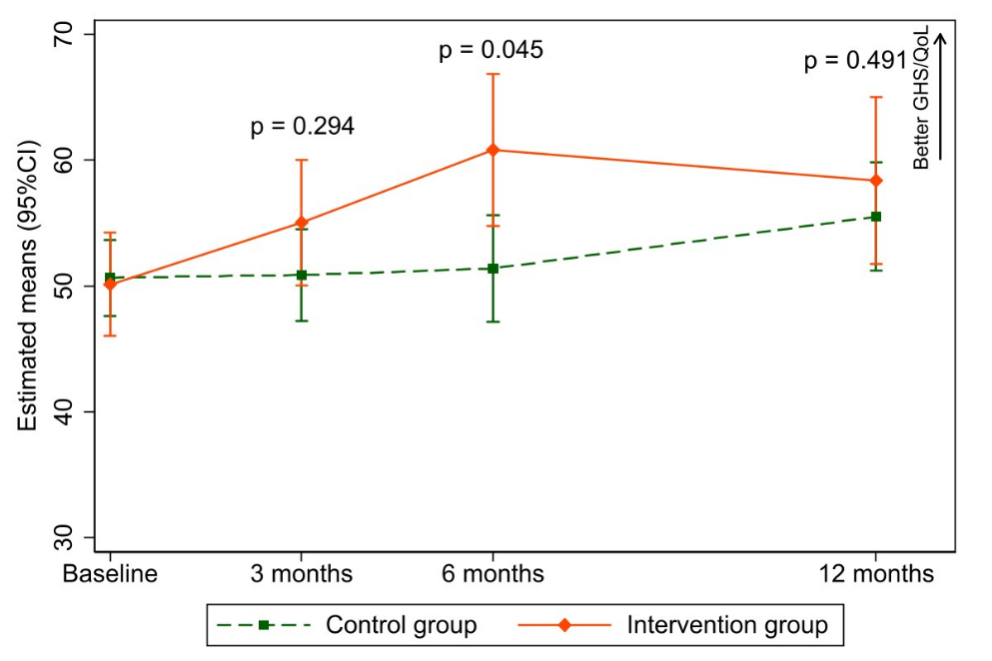
Changes in global health status/QoL (GHS/QoL) of the EORTC QLQ-C30 between groups. The possible range of GHS/QoL scores is 0-100, with higher values indicating better GHS/QoL. An IPTW-adjusted linear mixed model with multiple imputation (10 imputed data sets) and a random intercept for patients was performed. The model adjusted for baseline GHS/QoL scores, study site, follow-up times, group, and interaction between follow-up times and group as a fixed factor. Adjusted p-value using the Benjamini-Hochberg for controlling the false discovery rate for multiple comparison is presented. Abbreviations: CI, confidence interval; EORTC QLQ-C30, European Organisation for Research and Treatment of Cancer Quality of Life Questionnaire Core 30; QoL, Quality of Life; IPTW, Inverse Probability Treatment Weighting

At baseline the control group reported higher values in GHS/QoL than the intervention group (52.4 (21.7) vs. 47.6 points (21.8)). To balance the identified group differences at baseline and handle missing data due to dropout, we applied IPTW and imputed data using multiple imputation. After IPTW, there were no group differences at baseline (50.1 vs. 50.6 points) (Table 1). While the GHS/QoL scores in the control group remained almost unchanged after 3 and 6 months, the intervention group reported improved of the GHS/QoL after 3 months (mean scores of GHS/QoL: IG = 55.1 vs. CG = 50.9, mean difference = 4.2 points (95%CI: 2.7, 11.0), adjusted p-value = 0.294). The significant improvement of the GHS/QoL in the intervention group was observed after 6 months (IG = 60.8 vs. CG = 51.4, mean difference = 9.4 points (95%CI: 0.2, 18.7), adjusted p-value = 0.045). At 12 months, the GHS/QoL in the intervention group remained slightly above the level of the control group, but the groups differed only marginally (IG = 58.4 vs. CG = 55.5, mean difference of 2.9 points (95%CI: -5.4, 11.1; adjusted *p* = 0.491) (Fig. 2. Sensitivity analyses confirmed the robustness of the results and adequate addressing of missing data mechanisms (Online supplementary document Table S3).

#### Secondary outcomes

**Fig. 3:**
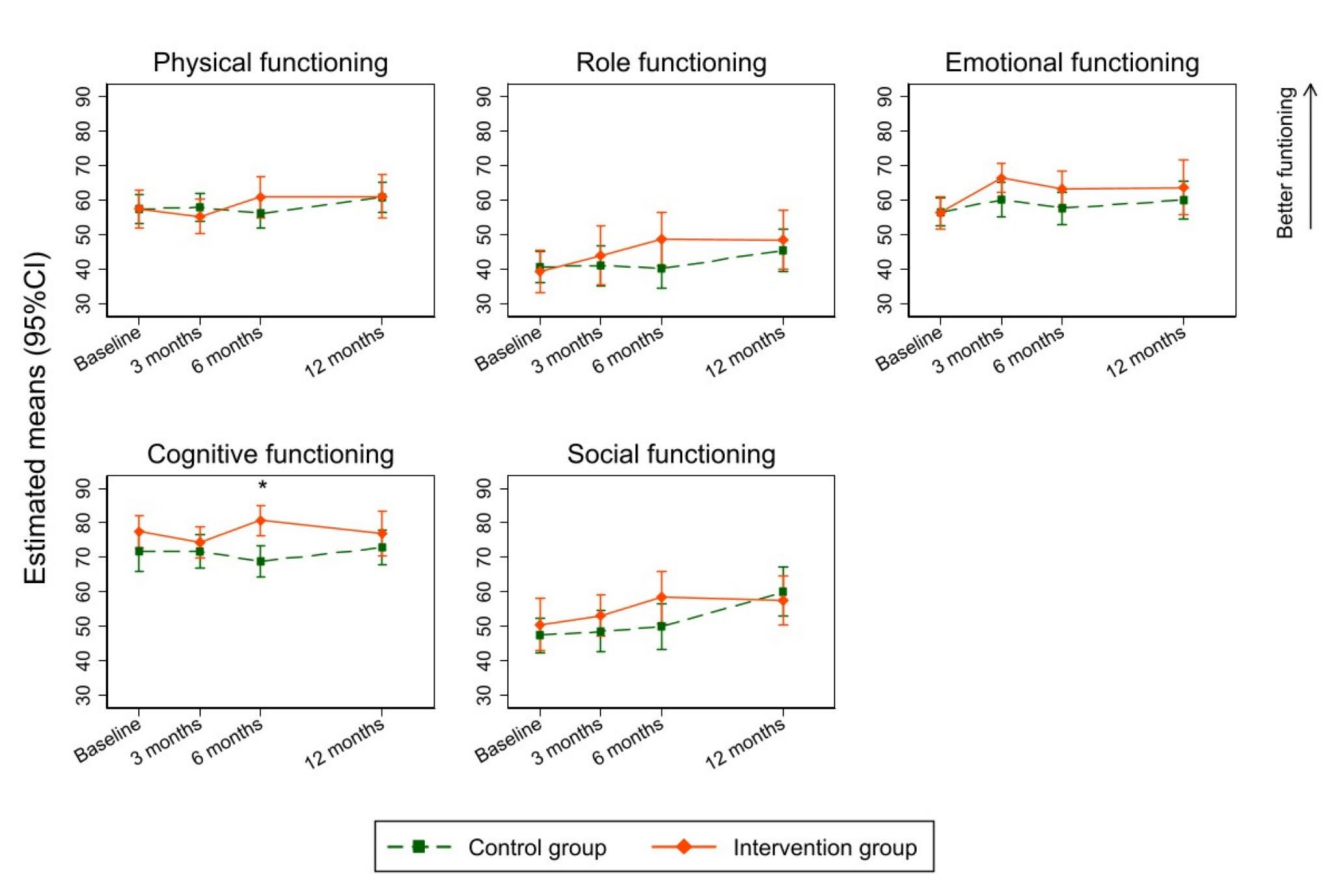
Changes in functional scales of the EORTC QLQ-C30 between groups. The possible range is 0-100, with higher values indicating better functioning. An IPTW-adjusted linear mixed model with multiple imputation (10 imputed data sets) and a random intercept for patients was performed. The model adjusted for baseline scores of each functioning domain, study site, follow-up times, group, and interaction between follow-up times and group as a fixed factor. * represents a statistically significant difference between groups in cognitive functioning at 6 months (intervention group = 80.7 vs. control group = 68.9, mean difference 11.8 (95%CI: 5.4, 18.2), p-value < 0.001). Abbreviations: EORTC QLQ C-30, European Organisation for Research and Treatment of Cancer Quality of Life Questionnaire Core 30; QoL, Quality of life. IPTW, Inverse Probability Treatment Weighting

**Fig. 4:**
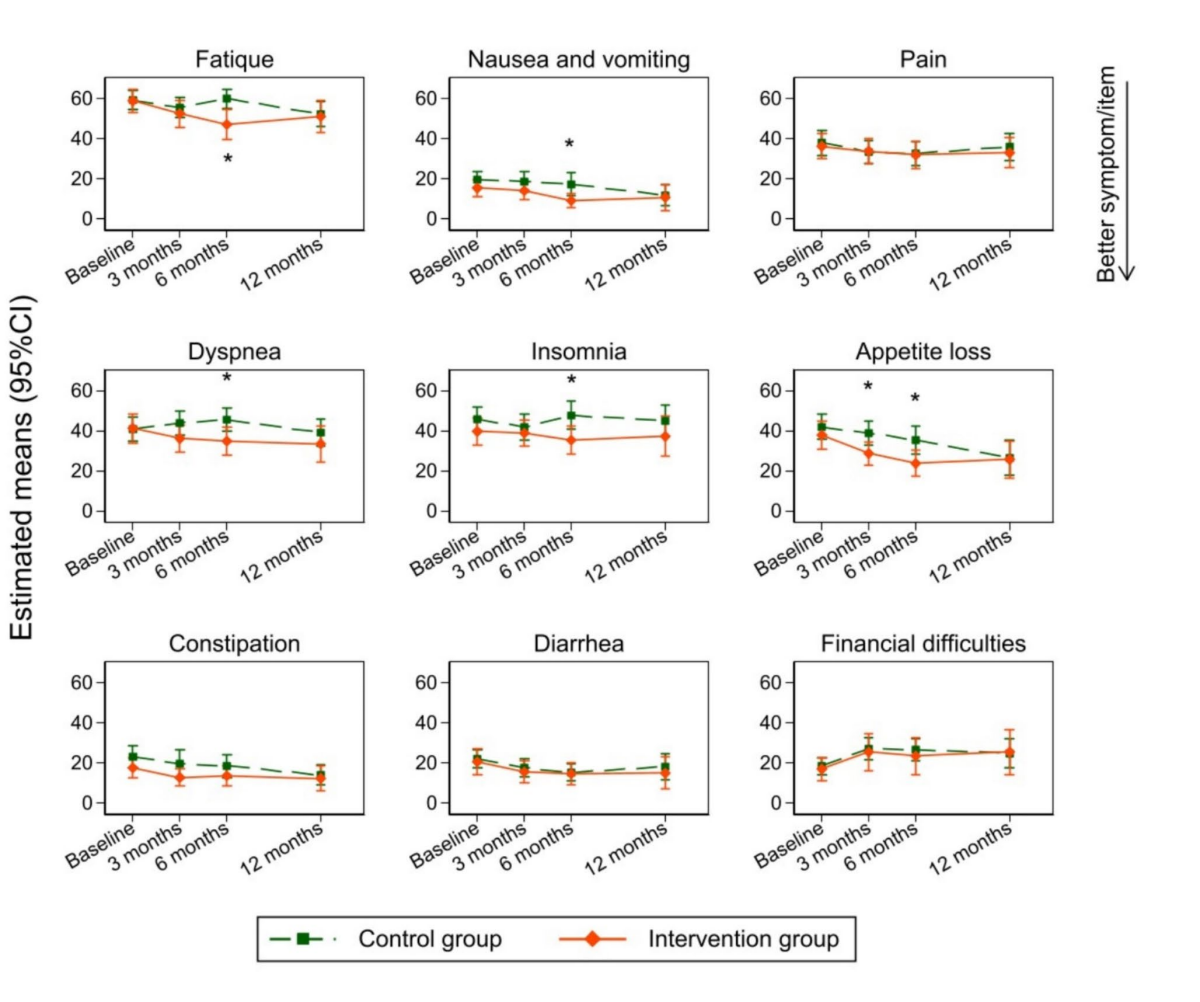
Changes in symptom and item scales of the EORTC QLQ-C30 between groups. The possible range is 0-100, with lower values indicating better symptom/item. An IPTW-adjusted linear mixed model with multiple imputation (10 imputed data sets) and a random intercept for patients was performed. The model adjusted for baseline scores of each symptom or item domain, study site, follow-up times, group, and interaction between follow-up times and group as a fixed factor. * represents a statistically significant difference between groups (p-value < 0.05). Abbreviations: CI, confidence interval; EORTC QLQ-C30, European Organisation for Research and Treatment of Cancer Quality of Life Questionnaire Core 30; QoL, Quality of Life; IPTW, Inverse Probability Treatment Weighting

With respect to secondary outcomes group differences were observed at baseline, we applied the same method as the primary outcome (IPTW with multiple imputation). Estimated means for functional and symptom subscales were more preferable in the intervention group compared to the control group over the entire observation period of 12 months (Figs. 3 and 4). The results indicate higher scores in cognitive functioning at 6 months in the intervention arm, with a mean difference between groups of 11.8 points (95%CI: 5.4, 18.2). Mean differences of 6 to 9 points were estimated in the scales of role functioning, emotional functioning, and social functioning after 6 months. Likewise after 6 months, patients in the intervention group showed improved scores in the symptom subscales relating to dyspnea (mean difference: -10.7 [95%CI: -19.7, -1.7]), insomnia (mean difference: -12.3 [95%CI: -22.3, -2.3]), fatigue (mean difference: -12.9 [95%CI: -21.7, -4.0]), nausea and vomiting (mean difference: -8.1 [95%CI: -14.8, -1.4]), and appetite loss (mean difference: -11.6 [95%CI: -21.5, -1.8]) (Online supplementary document, Table S4 and S5).

**Fig. 5:**
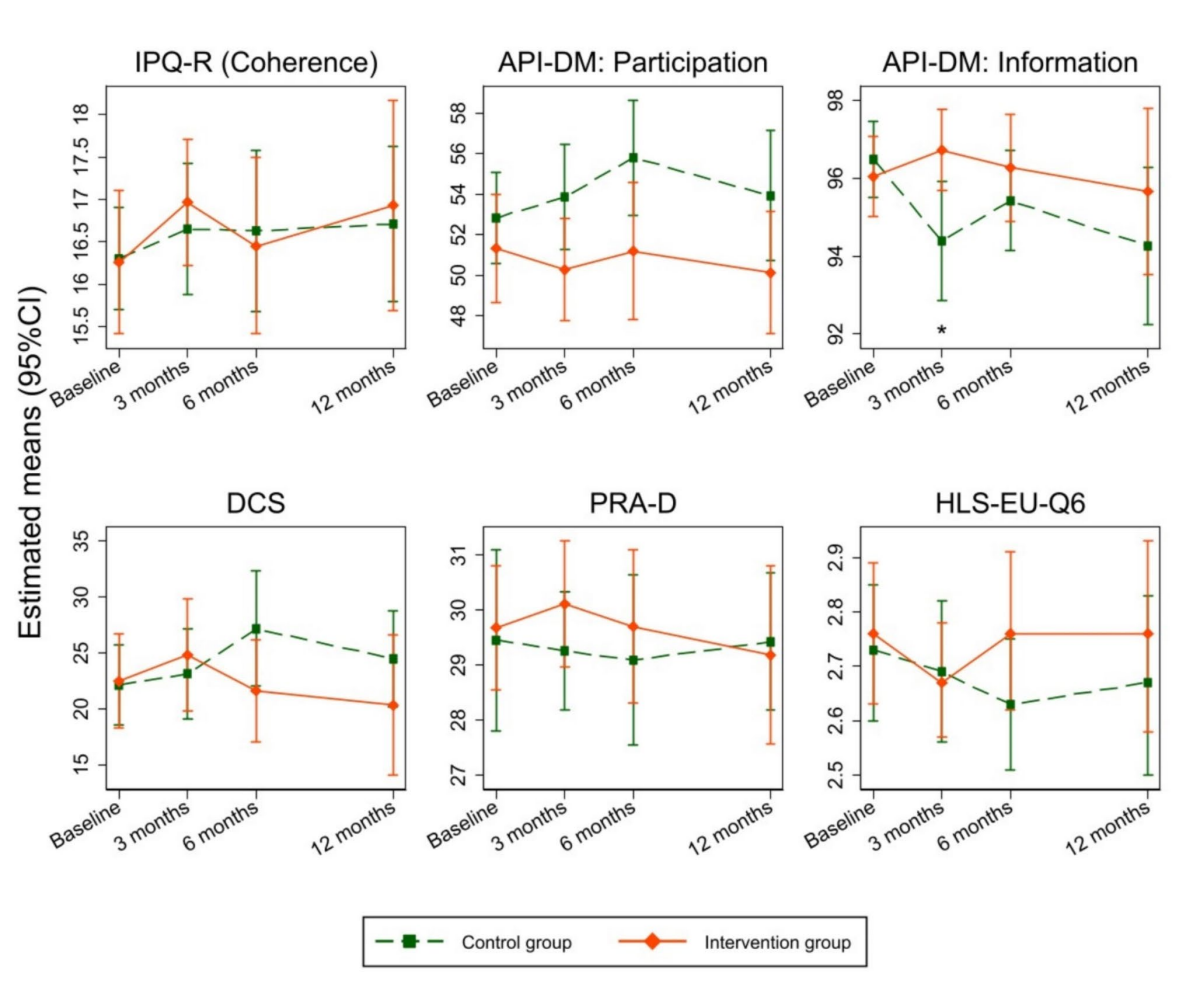
Changes in secondary outcomes over the study periods. High values of IPQ-R, API-DM: Information, PRA-D, and HLS-EU-Q6 indicate improvement or better outcome. Low values of DCS indicate less decision conflict in patient. The value of API-DM: Participation at 50 scores presents an equally participation between patient and physician. An IPTW-adjusted linear mixed model with multiple imputation (10 imputed data sets) and a random intercept for patients was performed. The model adjusted for baseline scores of each secondary outcomes, study site, follow-up times, group, and interaction between follow-up times and group as a fixed factor. * represents a statistically significant difference between groups in the preference for information of the API-DM (p-value = 0.012). Abbreviations: API-DM, German modified Version of the Autonomy Preference Index; CI, confidence interval; DCS, Decision Conflict Scale; HLS-EU-Q6, Health Literacy Questionnaire; IPQ-R, Illness-Perception-Questionnaire; PRA-D, German Version of the Patient Reactions Assessment; IPTW, Inverse Probability Treatment Weighting

After 12 months, patients in the intervention group showed more preferable scores on illness coherence (IPQ-R), perceived quality of patient-physician communication (PRA-D), decisional conflicts (DCS), information and participation preference (API-DM), and health literacy (HLS-EU-Q6) compared to the control group (Fig. 5). At 3 months, API-DM subscale scores revealed vast group differences in information preferences (mean difference: 2.3 [95%CI: 0.5, 4.1]) and participation preferences (mean difference: -3.6 [95%CI: -7.3, 0.1]). Further indications of group differences were found in participation preferences, DCS, and HLS-EU-Q6 at six months (online supplementary document, Table S6). However, these differences were minor.

## Discussion

Our findings demonstrate that monthly cross-sectoral navigational support and coordinating psychosocial counselling by SCNs results in an improvement in GHS/QoL scores after 3 and 6 months in patients with advanced cancer. The intervention group developed more favorably than the control group on secondary endpoints, such as better cognitive functioning and a reduction of symptom burden in five out of nine subscales at 6 months. Regarding information preferences, group differences in favor of the intervention were observed after 3 months. After 12 months, the observed group differences disappeared.

### Improvement in GHS/QoL

The very low QoL scores reported over the observation period emphasize the vulnerability of the target population as well as the relevance of these types of interventions. The scores are comparable to the QoL of palliative oncology patients in their last year of life [48–50]. Regarding reference scores for the change in EORTC as defined by Cocks et al., the observed change in global health status/QoL after six months represents a medium improvement in the intervention arm [51–53]. Vanbutsele et al.’s study showed a comparable, although a little earlier, benefit for GHS/QoL with an early and systematic palliative care approach led by palliative care nurses. Whereby the focus here was also primarily on inpatient care [50]. A first explanation for the increased QoL [8, 14] and decreased symptom burden [16, 54, 55] might be the psycho-oncological components of the intervention. Previous findings suggest that patients benefit especially from this type of support service. It should be noted that the primary role of the SCNs was to coordinate psycho-oncology services rather than to provide them. Nonetheless, the majority of the six SCNs had additional training in psycho-oncology. In contrast, programs focusing merely on patient navigation have shown to have little or no impact on quality of life [23, 25, 56, 57]. A second explanation for the higher QoL could be the additional time for care (monthly consultations) and the more personal interview situation offered by SCNs in which patients might express their needs in more detail [7, 50]. The significantly longer duration of care of 12 months and the proactivity of the SCN compared to other studies also seem to contribute to the increase in QoL. Faller et al. reported in a systematic review of 198 studies that psycho-oncologic interventions require long (continuous) treatments and are particularly effective when they go beyond the mere provision of information [8]. Furthermore, our results support previous study findings describing the correlation between the effectiveness of the intervention and the qualification of the staff offering navigational services. Patients benefited more likely from navigational interventions delivered by cancer experienced and professional medical staff in terms of quality of life [21, 23, 25, 57].

To facilitate the use of PROMs in daily clinical practice and research, thresholds for clinical importance (TCI) are highly useful, as they enable a more patient-oriented interpretation of the metric scales and therefore make the data more accessible and immediately usable for practice [49]. With regard to previously established thresholds for the EORTC QLQ-C30 to identify clinically important problems or symptoms, there were positive changes in our intervention group. After six months, both study arms remained severely affected but the scores for cognitive and social functioning were now no longer in the range for clinical importance reported by Giesinger et al. [49]. Further positive trends were observed in the symptom scales for nausea and vomiting, where a strong approach towards the threshold value could be observed. For insomnia, both groups tended to report values below the threshold, but the intervention patients carried a lower symptom burden, well below the TCI. For diarrhea, patients in the intervention reported values below the TCI after 3 and 12 months compared to patients in the control group [49].

### Importance of changes in further secondary outcomes

Differences between the intervention and control groups were observed in secondary outcomes too, with positive results for the intervention group during single follow-up assessments.

Participation and information preferences in both groups illustrate the practice of joint decision-making with the treating physician and a high need for information on diagnosis and therapy, with preferences being better addressed in the intervention group already after 3 months. In a French validation study of API instruments and their adaptation for oncology patients, with a comparable study population, lower scores were found for both preferences [58].

Both groups showed generally low potential for decisional conflicts (DCS), with the strongest difference identifiable in the intervention group after six months. In a small US validation study of the instrument with patients with prostate cancer, higher potential for decisional conflicts were reported [59]. The results complement previous research on decision-making conflicts among severely ill patients and suggest that, for example, the trade-off between quality of life vs length of life, which often leads to decision-making conflicts, is rather not negotiated in the group, similarly to older people, for whom the conflicts occur less frequently due to shorter life expectancy and high comorbidity [60]. The intervention groups’ increases in patient-physician communication and health literacy values were slightly higher than the control groups’. The values are comparable with previously published values [61]. However, even minor changes in health literacy play a special role in the treatment of the severely ill, as patients with increased competence verbalize their own needs more often [7] and show an increased probability to take up navigation services [14].

### Minor differences for the entire observation period

After 12 months, the inter-group difference in QoL disappeared. The results are thus comparable with previous findings [8]. Within-group changes indicate medium improvements in global health/QoL in the intervention group and small increases in the control group [51]. The observed group alignments might be explained due to death and deterioration in health status which particularly affected the intervention patients, suggesting higher vulnerability after recruitment. Other possible explanations include a response shift in patients answering the questionnaires repeatedly, ongoing toxicity, or additional health care utilization during the observation period [47]. QoL reported at 12 months is similar to levels reported in intervention studies with severely ill patients [50]. Taking the long observation period into account, effects of informal transmission of the intervention material could be an explanation for the convergence of the two groups. Another possible explanation is that patients in the control group might have received care from SCNs, albeit with lower frequency. Furthermore, patients were recruited in hospitals where specialized oncology nurses, psycho-oncologists and social workers are routinely part of the team and get regularly involved over time, especially as the disease progresses.

The pattern of change in QoL over time suggests that the intervention has a catalytic effect. Patients with continuous support from an SCN achieved higher QoL twice as quickly, an effect that should not be underestimated given the life expectancy of the target population [47]. In view of the positive trends identified, the Innovation Fund of the German Federal Joint Committee (funder) forwarded the results of our study to the German Cancer Society and the associations of health and long-term care insurance funds at federal level. The institutions are asked to examine whether the approaches of the new services can be developed further and implemented into statutory treatment. In order to provide SCN services to all patients, the financial and human resources would have to be significantly increased compared to the study phase. Utilization of the service was very heterogeneous. While the minimum monthly contact was sufficient for some patients, other patients or their relatives contacted the SCN weekly or even at night. The programme appears to be distinct from existing services, particularly in terms of long-term cross-sectoral staff continuity, that does not end with discharge from hospital.

### Limitations

Randomization of the observation groups was not feasible due to the specific funding framework in combination with the specific inclusion criteria. Recruitment-related selection bias was exposed through a previously published comparative analysis of baseline characteristics [32]. Adjustments for baseline unbalance between study groups were applied by the IPTW method. While multiple imputation procedures were used due to the premature dropout of half of all recruited participants, some limitations should be acknowledged. Multiple imputation assumes that data are MAR and might provide biased results if the data are MNAR. For this reason, we did not apply the imputation method to patient who died early, as unobserved assessments after death will not be considered missing data [12]. Additionally, the accuracy of the imputed values depends on the correct specification of the imputation model, as any misspectification could lead to biased results. Given the high missing rate observed in our study, the accuracy of the multiple imputation model is critical and could lead to high variability in the imputed datasets, affecting the final estimates. Despite these limitations, we have taken careful steps to validate our imputation model and performed various sensitivity analyses to ensure a robust framework for addressing missing data. However, similarly high dropout rates were also seen in other studies in oncology [62–64]. Patient group participation was not blinded to physicians and clinical staff, which is assumed to have had no impact on treatment and communication. Moreover, the inclusion of heterogeneous oncological entities makes a differentiated assessment difficult and impedes the generalizability of the results. Previous studies showed differences in supportive care needs between tumor types. Furthermore, we could not consider information about cancer staging, receiving palliative care, and other supportive care services in our analysis. Finally, it should be noted that between the third and fourth observation point there was a comparatively long period of six months and therefore the development of the quality of life cannot be depicted in detail. In future, a further survey at nine months could provide more information.

## Conclusion

Over the study period of one year, our analyses demonstrate that patients with advanced cancer could benefit from navigational support provided by the SCNs regarding their quality of life, especially in the first six months after initiation of the support. Strengths of the service include continuity of care, the structured questionnaire-based identification of care needs and coordination of psychosocial support. In the future, regular digital assessments could make progression or degression visible more timely; these could also be made available to the medical professions involved.

## Electronic supplementary material

Below is the link to the electronic supplementary material.

Supplementary Material 1

Supplementary Material 2
