# Supplementary material for: The effect of social care nurses on health related quality of life in patients with advanced cancer: A non-randomized, multicenter, controlled trial
Source: Qual Life Res. 2024 Sep 13;33(12):3387–99. doi: 10.1007/s11136-024-03780-3 (PMC11599374; doi:10.1007/s11136-024-03780-3)
Supplement: Supplementary file 2 — Supplementary Material 2 [file 11136_2024_3780_MOESM2_ESM.docx]

**Online Resource - Supplementary material**

**The effect of social care nurses on health related quality of life in patients with advanced cancer: A non-randomized, multicenter, controlled trial**

Daniel Schindel^1^, Johann Frick^1^, Pimrapat Gebert^2,3^, Ulrike Grittner^2,3^, Anne Letsch^4,5^, and Liane Schenk^1^

^1^ Charité – Universitätsmedizin Berlin, corporate member of Freie Universität Berlin, Humboldt-Universität zu Berlin, and Berlin Institute of Health, Institute of Medical Sociology and Rehabilitation Science, Charitéplatz 1, 10117 Berlin, Germany

^2^ Charité – Universitätsmedizin Berlin, corporate member of Freie Universität Berlin, Humboldt-Universität zu Berlin, and Berlin Institute of Health, Institute of Biometry and Clinical Epidemiology, Charitéplatz 1, 10117 Berlin, Germany

^3^ Berlin Institute of Health (BIH), Anna-Louisa-Karsch-Str. 2, 10178 Berlin, Germany

^4^ University Hospital Schleswig-Holstein, Department of Medicine II, Hematology and Oncology, Arnold-Heller-Straße 3, 24105 Kiel, Germany

^5^ Charité – Universitätsmedizin Berlin, corporate member of Freie Universität Berlin, Humboldt-Universität zu Berlin, and Berlin Institute of Health, Charité Comprehensive Cancer Center, Charitéplatz 1, 10117 Berlin, Germany

**Statistical analysis**

***Comparative baseline analysis***

The inverse probability of treatment weighting (IPTW) method was applied for controlling imbalanced baseline characteristics between the intervention and control groups. The weighting propensity scores were estimated by a logistic regression model; the covariates included are presented in Table 2 and Figure 6 (see below). The standardized mean difference (SMD) was calculated to check the balancing of baseline characteristics before and after IPTW adjustment. Figure 6 presents the SMD before and after IPTW adjustment for each baseline characteristic. After IPTW adjustment, baseline characteristics between the intervention and control group were balanced, with SMD<+/-0.1 for all adjusted characteristics.

***Missing data***

*Missing on baseline characteristics*

Missing data is present less than 10% for family status, care level, duration of living in Germany, education, financial situation, subjective social status, and social support (Table 1).

*Missing of patient reported outcome measures (PROMs)*

Approximately half of the patients dropped out before the study end (after 12 months), with a median follow-up time of 333 days (IQR: 154, 361 days). 169 patients (46.7%) did not complete the EORTC QLQ-C30 questionnaire (the missing rate for the intervention and control group was 46.0% and 47.2%, respectively) and 173 patients (47.8%) did not complete the other PROMs (the missing rate for the intervention and control group was 48.7% and 47.2%, respectively) at all four assessment times/ points. Most of the missing EORTC QLQ-C30 values were related to premature death (28.0% (42/150) in the intervention group and 28.3% (60/212) in the control group), while 12% of patients (11.3% (17/150) in the intervention group and 12.7% (27/212) in the control group) dropped out for health-related reasons.

***Missing imputation***

Multiple imputations with chained equations (MICE) were used to handle missing data that were assumed to be missing at random (MAR) for all covariates. The missing values for patients who died during the study period were not imputed, since missing not at random (MNAR) is assumed. The missing data at each time point were imputed, stratified by study groups. We conducted MICE using predictive mean matching regression for the missing elements within the EORTC QLQ-C30, IPQ-R, PRA-D, DCS, API-DM, HLS-EU-Q6 and subjective social support, ordinal logistic regression for education, and multinomial logistic regression for family status. Auxiliary variables in the imputation model were study group, age, sex, study site, follow-up time, diagnosis, time since diagnosis, and reasons for missing (death, health-related drop-out, non-health-related drop-out). We generated ten imputed data sets [1] and summarized the effect estimates and variances using Rubin’s rules [2].

***Sensitivity analysis***

Missing values for the global health status/QoL (GHS/QoL) scale of the EORTC QLQ-C30 might not missing at random (MNAR) due to dropout and death. After having imputed missing data for all cases including death by means of MAR assumption, we modified the global health status/QoL scores by rescaling [3, 4]. Based on our data, we assumed that the GHS/QoL in the dropout patients could be up to 5% and 10% lower for the intervention group and the control group, respectively. For values missing due to death, the GHS/QoL scores could be up to 10% and 20% lower for the control group and 20% and 25% lower for the intervention group. We conducted two additional sensitivity analyses, taking into account missing data due to death. First, we used a joint model which combines two sub-models to analyze longitudinal and survival outcomes [5]. The first sub-model was the IPTW-adjusted linear mixed model as described above. The second, a Cox regression model, compared the survival outcomes between groups adjusted for the study site. Second, assuming death patients had the worst score of the GHS/QoL (the zero score is given to the patients at the time they died), and the IPTW-adjusted linear mixed model as described before was performed. The level of significance was set to 0.05 (two-sided test). All statistical tests were performed using Stata IC15 (StataCorp, 2017, College Station, TX, USA).

**
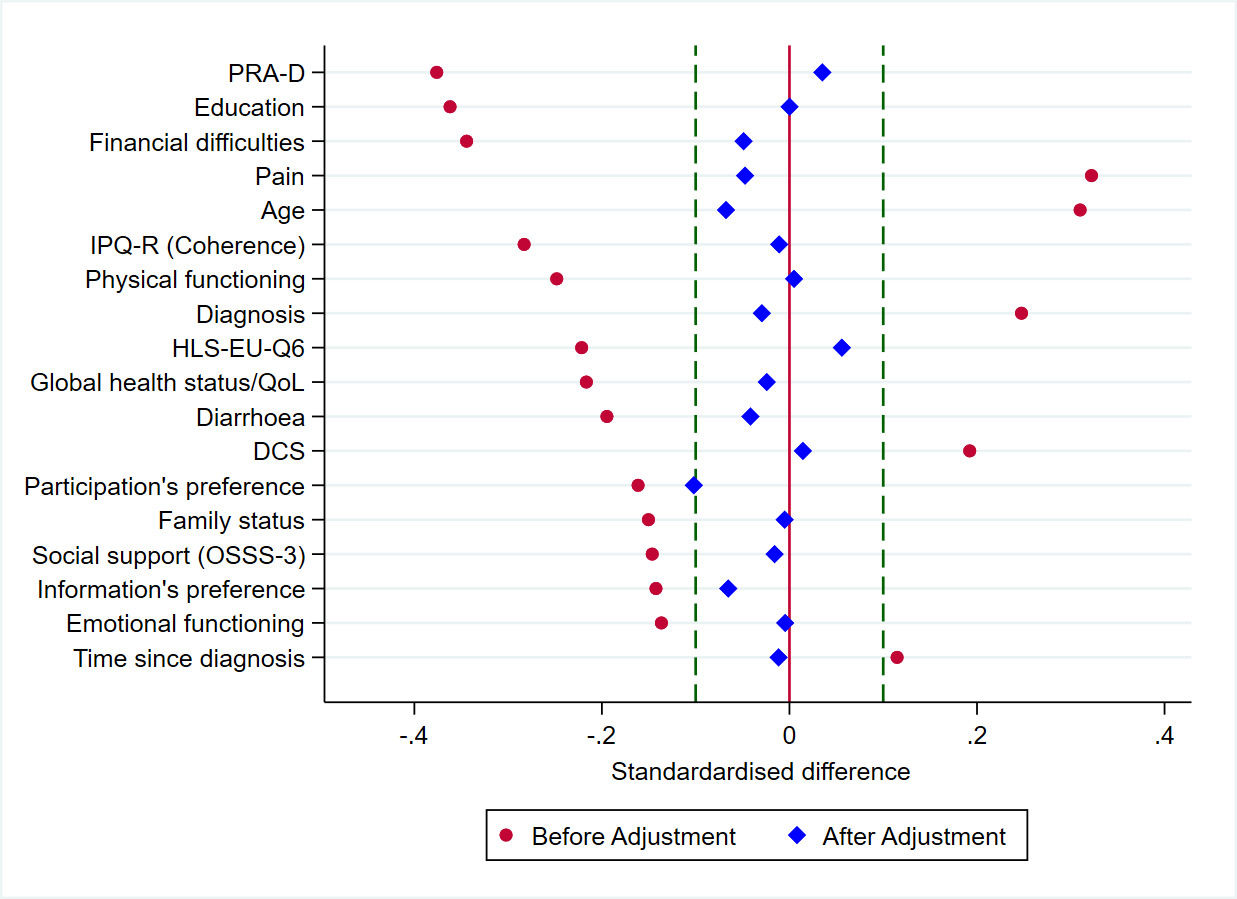
**

**Figure S1** Balancing before and after adjustment by Inverse Probability of Treatment Weighting (IPTW) on the baseline characteristics between intervention group and control group.
Abbreviations: DCS, Decision Conflict Scale; HLS-EU-Q6, Health Literacy Questionnaire; IPQ-R, Illness-Perception-Questionnaire; IPTW, inverse probability of treatment weighting; Osss-3, Oslo social support scale; PRA-D, German Version of the Patient Reactions Assessment; QoL, Quality of Life.

| **ICD-Codes with 5 digits (summary)** |
| --- |
| 1. Metastasized malignant neoplasms of lip, oral cavity, and pharynx   (C00 – C14 and C77 – C79) |
| 1. Malignant neoplasm of esophagus   (C15) |
| 1. Malignant neoplasm of stomach   (C16) |
| 1. Metastatic colorectal cancer / colon carcinoma   (C18 – C20 and C77 – C79) |
| 1. Malignant neoplasm of pancreas   (C25) |
| 1. Malignant neoplasm of bronchus and lung   (C34) |
| 1. Metastasized melanoma and other malignant neoplasms of skin   (C43 – C44 and C77 – C79) |
| 1. Metastasized malignant neoplasm of breast   (C50 and C77 – C79) |
| 1. Metastasized malignant neoplasm of ovary   (C56 and C77 – C79) |
| 1. Metastasized malignant neoplasm of cervix uteri   (C53 and C77 – C79) |
| 1. Metastasized malignant neoplasm of corpus uteri (C54 and C77 – C79) |
| 1. Metastasized malignant neoplasm of prostate (C61 and C77 – C79) |
| 1. Metastasized malignant neoplasm of thyroid gland  (C73 and C77 – C79) |
| 1. Lymphoma  (C82 – C86) |
| 1. Multiple myeloma and malignant plasma cell neoplasms (C90) |
| 1. Leukemia  (C91 – C92) |
| **OPS-Codes (summary)** |
| 1. Surgical operation on the digestive tract   (5-42 – 5-54) |
| 1. Surgical operation on the lymphatic tissues   (5-402 – 5-404; 5-406) |
| 1. Radiotherapy, nuclear medicine therapy and pain management   (8-52; 8-53; 8-91) |
| 1. Multimodal pain treatment, cytotoxic chemotherapy, complex treatment   (8-541 – 8-544; 8-546; 8-918; 8-982; 8-98e) |

**Table S1** Inclusion criteria for the intervention and control group.
Abbreviations: ICD, International Classification of Diseases; OPS, Coding’s for medical surgeries and procedures in Germany.

**Table S2** Descriptive statistics for primary and secondary outcomes for each follow-up time before imputation

| **Primary and secondary outcomes** | **Time** | **Control** | | **Intervention** | |
| --- | --- | --- | --- | --- | --- |
|  |  | **n** | **Mean (SD)** | **n** | **Mean (SD)** |
| global health status/QoL (GHS/QoL) | Baseline | 208 | 52.6 (21.7) | 147 | 47.8 (22.0) |
|  | 3-month | 160 | 54.1 (22.8) | 116 | 52.8 (22.6) |
|  | 6-month | 137 | 55.7 (23.3) | 91 | 57.3 (23.4) |
|  | 12-month | 113 | 58.4 (22.1) | 78 | 59.2 (26.0) |
| Physical functioning (PF) | Baseline | 209 | 60.2 (25.9) | 149 | 54.0 (24.3) |
|  | 3-month | 159 | 61.6 (25.4) | 116 | 52.5 (26.7) |
|  | 6-month | 137 | 60.9 (25.7) | 94 | 57.6 (28.1) |
|  | 12-month | 113 | 65.3 (27.3) | 78 | 60.0 (29.1) |
| Role functioning (RF) | Baseline | 212 | 41.7 (33.7) | 147 | 39.0 (33.8) |
|  | 3-month | 160 | 44.7 (32.9) | 114 | 39.8 (34.4) |
|  | 6-month | 137 | 43.9 (32.1) | 93 | 47.0 (32.1) |
|  | 12-month | 113 | 49.7 (32.1) | 76 | 52.0 (34.1) |
| Role functioning (RF) | Baseline | 208 | 58.7 (27.6) | 149 | 55.0 (26.3) |
|  | 3-month | 160 | 62.5 (28.4) | 116 | 63.4 (25.7) |
|  | 6-month | 137 | 59.5 (27.9) | 94 | 64.2 (24.6) |
|  | 12-month | 113 | 61.4 (27.4) | 78 | 65.4 (27.8) |
| Cognitive functioning (CF) | Baseline | 209 | 72.8 (30.4) | 149 | 75.5 (27.2) |
|  | 3-month | 160 | 71.2 (28.2) | 116 | 73.9 (27.0) |
|  | 6-month | 136 | 69.7 (28.7) | 94 | 80.5 (25.5) |
|  | 12-month | 113 | 72.3 (27.6) | 78 | 76.7 (26.6) |
| Social functioning (SF) | Baseline | 208 | 48.4 (35.6) | 149 | 51.6 (34.4) |
|  | 3-month | 159 | 48.8 (34.1) | 114 | 53.4 (33.4) |
|  | 6-month | 137 | 52.2 (33.3) | 94 | 58.2 (32.2) |
|  | 12-month | 113 | 60.2 (32.7) | 78 | 60.9 (32.9) |
| Fatigue (FA) | Baseline | 211 | 56.6 (29.5) | 148 | 59.3 (28.2) |
|  | 3-month | 160 | 53.9 (31.7) | 116 | 56.6 (31.2) |
|  | 6-month | 137 | 56.4 (30.0) | 94 | 49.7 (29.6) |
|  | 12-month | 113 | 49.8 (29.0) | 78 | 50.4 (33.0) |
| Nausea and vomiting (NV) | Baseline | 212 | 17.9 (26.5) | 148 | 16.7 (27.1) |
|  | 3-month | 160 | 17.7 (28.4) | 116 | 15.4 (25.4) |
|  | 6-month | 137 | 15.8 (24.6) | 94 | 9.8 (20.7) |
|  | 12-month | 113 | 11.1 (19.4) | 78 | 8.3 (18.6) |
| Pain (PA) | Baseline | 212 | 32.5 (35.0) | 149 | 44.1 (36.8) |
|  | 3-month | 160 | 30.2 (32.0) | 116 | 39.9 (35.8) |
|  | 6-month | 137 | 30.5 (31.3) | 94 | 36.9 (36.0) |
|  | 12-month | 113 | 30.5 (32.1) | 78 | 34.6 (33.3) |
| Dyspnoea (DY) | Baseline | 211 | 38.2 (37.7) | 149 | 40.9 (39.2) |
|  | 3-month | 160 | 40.2 (35.3) | 114 | 40.6 (34.0) |
|  | 6-month | 136 | 40.9 (36.3) | 92 | 39.5 (32.4) |
|  | 12-month | 112 | 35.1 (33.4) | 78 | 37.2 (33.1) |
| Insomnia (SL) | Baseline | 211 | 45.5 (38.8) | 149 | 41.6 (39.1) |
|  | 3-month | 160 | 43.1 (34.6) | 115 | 40.0 (35.4) |
|  | 6-month | 137 | 45.5 (36.8) | 94 | 34.8 (35.6) |
|  | 12-month | 113 | 43.7 (36.5) | 78 | 33.8 (34.2) |
| Appetite loss (AP) | Baseline | 212 | 38.8 (38.5) | 147 | 40.8 (40.1) |
|  | 3-month | 160 | 36.2 (36.3) | 116 | 30.2 (35.7) |
|  | 6-month | 137 | 32.1 (36.9) | 93 | 23.3 (29.4) |
|  | 12-month | 113 | 21.5 (30.5) | 78 | 21.8 (29.3) |
| Constipation (CO) | Baseline | 211 | 22.1 (34.5) | 149 | 21.5 (32.9) |
|  | 3-month | 159 | 19.9 (32.3) | 115 | 15.7 (27.7) |
|  | 6-month | 137 | 18.0 (30.3) | 94 | 15.2 (27.1) |
|  | 12-month | 113 | 15.9 (26.8) | 78 | 14.1 (27.7) |
| Diarrhoea (DI) | Baseline | 212 | 24.5 (33.7) | 149 | 18.1 (30.9) |
|  | 3-month | 159 | 18.4 (28.5) | 116 | 17.0 (30.6) |
|  | 6-month | 136 | 16.9 (27.2) | 94 | 14.5 (27.5) |
|  | 12-month | 113 | 20.4 (28.0) | 77 | 12.1 (23.5) |
| Financial difficulties (FI) | Baseline | 208 | 22.1 (33.9) | 149 | 12.1 (23.6) |
|  | 3-month | 160 | 28.1 (35.4) | 115 | 18.0 (27.7) |
|  | 6-month | 137 | 29.0 (33.3) | 94 | 16.7 (25.3) |
|  | 12-month | 113 | 25.4 (34.0) | 77 | 16.9 (28.9) |
| IPQ-R | Baseline | 209 | 16.6 (4.5) | 123 | 15.6 (4.0) |
|  | 3-month | 160 | 17.1 (4.1) | 96 | 16.6 (4.2) |
|  | 6-month | 137 | 17.0 (4.8) | 85 | 16.0 (4.6) |
|  | 12-month | 112 | 17.2 (4.8) | 66 | 17.0 (4.3) |
| PRA-D | Baseline | 211 | 30.6 (6.0) | 124 | 28.4 (7.0) |
|  | 3-month | 160 | 29.9 (6.0) | 97 | 29.6 (6.7) |
|  | 6-month | 137 | 29.9 (6.4) | 84 | 29.9 (5.9) |
|  | 12-month | 113 | 30.3 (6.1) | 66 | 29.8 (6.6) |
| API-DM: Preference for participation | Baseline | 209 | 53.7 (15.3) | 127 | 51.5 (14.1) |
|  | 3-month | 160 | 55.4 (15.9) | 97 | 49.8 (13.2) |
|  | 6-month | 138 | 54.9 (15.2) | 84 | 50.6 (12.2) |
|  | 12-month | 113 | 53.4 (14.6) | 66 | 49.2 (12.7) |
| API-DM: Preference for information | Baseline | 210 | 96.5 (6.0) | 127 | 95.4 (7.3) |
|  | 3-month | 160 | 94.5 (8.8) | 97 | 96.4 (5.7) |
|  | 6-month | 138 | 95.4 (7.6) | 84 | 96.5 (6.1) |
|  | 12-month | 112 | 94.2 (8.0) | 66 | 96.4 (5.7) |
| DCS | Baseline | 211 | 22.1 (24.8) | 124 | 26.2 (24.2) |
|  | 3-month | 159 | 22.2 (22.8) | 96 | 27.4 (25.3) |
|  | 6-month | 137 | 26.1 (25.3) | 80 | 22.9 (22.7) |
|  | 12-month | 113 | 24.7 (23.9) | 65 | 20.2 (20.0) |
| HLS-EU-Q6 | Baseline | 164 | 2.8 (0.7) | 79 | 2.6 (0.6) |
|  | 3-month | 123 | 2.7 (0.6) | 72 | 2.6 (0.6) |
|  | 6-month | 106 | 2.6 (0.6) | 67 | 2.7 (0.6) |
|  | 12-month | 94 | 2.7 (0.5) | 53 | 2.7 (0.7) |

IPQ-R=Illness Perception Questionnaire, PRA-D=Patient Reaction Assessment, API-DM=German modified version of the Autonomy Preference Index, DCS=Decisional Conflict Scale, HLS-EU-Q6=European Health Literacy Survey

| **Change in global health status/QoL (GHS/QoL)** |  | **Follow-up visits** | | |
| --- | --- | --- | --- | --- |
|  | **Baseline**  **(n = 362)** | **3 months**  **(n = 313)** | **6 months**  **(n = 289)** | **12 months**  **(n = 260)** |
| **Linear mixed model^#^** |  |  |  |  |
| Intervention group – estimated mean (95%CI) | 50.1 (46.0, 54.2) | 55.1 (50.1, 60.0) | 60.8 (54.8, 66.7) | 58.4 (51.8, 65.0) |
| Control group – estimated mean (95%CI) | 50.6 (47.6, 53.7) | 50.9 (47.3, 54.5) | 51.4 (47.2, 55.6) | 55.5 (51.2, 59.8) |
| Mean difference (IG-CG) – estimated mean (95%CI) | -0.5 (-5.6, 4.6) | 4.2 (-2.7, 11.0)* | 9.4 (0.2, 18.7) * | 2.9 (-5.4, 11.1) |
| Unadjusted p-value | 0.840 | 0.196 | 0.015 | 0.491 |
| Adjusted p-value* |  | 0.294 | 0.045 | 0.491 |
| **Sensitivity analysis** | **Baseline**  **(n = 362)** | **3 months**  **(n = 362)** | **6 months**  **(n = 362)** | **12 months**  **(n = 362)** |
| **Pattern-mixture model^##^** |  |  |  |  |
| ***Drop-out (IG=5%, CG=10%) Dead (IG=25%, CG=20%)*** |  |  |  |  |
| Mean difference (IG-CG) – estimated mean (95%CI) |  | 4.0 (-1.7, 9.6) | 7.5 (0.79, 14.2) | 3.4 (-3.4, 10.2) |
| p-value |  | 0.167 | 0.028 | 0.327 |
| ***Drop-out (IG=5%, CG=10%) Dead (IG=20%, CG=10%)*** |  |  |  |  |
| Mean difference (IG-CG) – estimated mean (95%CI) |  | 3.5 (-2.2, 9.1) | 6.9 (0.16, 13.6) | 2.3 (-4.5, 9.1) |
| p-value |  | 0.224 | 0.045 | 0.498 |
| **Joint models** |  |  |  |  |
| Mean difference (IG-CG) – estimated mean (95%CI) |  | 4.5 (-2.1, 11.1) | 9.7 (1.8, 17.7) | 2.8 (-6.0, 11.6) |
| p-value |  | 0.185 | 0.016 | 0.528 |
| **Worst case scenario (Zero score for death patients)** |  |  |  |  |
| Mean difference (IG-CG) – estimated mean (95%CI) |  | 4.5 (-2.1, 11.1) | 8.9 (1.1, 16.7) | 8.4 (-0.005, 16.7) |
| p-value |  | 0.178 | 0.026 | 0.050 |

**Table S3** Change in global health status/QoL (GHS/QoL) in the EORTC QLQ-C30.
Note: The pooled mean and 95%CI was estimated based on the IPTW-adjusted linear mixed models with multiple imputation (10 imputed data sets) and a random intercept for patients. All models adjusted for baseline GHS/QoL scores, study site, follow-up times, group, and interaction between follow-up times and group as a fixed factor. n = number of cases.
*p-values and confidence intervals were adjusted by Benjamini- Hochberg method, ^#^Patients who died at that follow-up time were not imputed. ^##^The GHS /QoL scores were modified by rescaling.
Abbreviations: CG, Control group; EORTC QLQ-C30, European Organisation for Research and Treatment of Cancer Quality of Life Questionnaire Core 30; IG, Intervention group; QoL, Quality of Life.

| **Functional scales of the EORTC QLQ-C30** | **Intervention group (IG)**  **Mean (95%CI)** | **Control group (CG)**  **Mean (95%CI)** | **Mean difference (IG–CG)**  **(95% CI)** | **p-value** |
| --- | --- | --- | --- | --- |
| **Physical functioning (PF)** |  |  |  |  |
| Baseline | 57.6 (52.1, 63.1) | 57.5 (53.3, 61.6) | 0.1 (-6.7, 7.0) | 0.972 |
| 3-month | 55.3 (50.2, 60.4) | 58.0 (53.8, 62.1) | -2.7 (-9.1, 3.7) | 0.409 |
| 6-month | 60.9 (54.9, 66.9) | 56.2 (51.9, 60.5) | 4.6 (-2.5, 11.8) | 0.202 |
| 12-month | 61.1 (54.8, 67.3) | 60.8 (56.4, 65.3) | 0.2 (-7.4, 7.9) | 0.944 |
| **Role functioning (RF)** |  |  |  |  |
| Baseline | 39.4 (33.3, 45.5) | 40.6 (36.0, 45.3) | -1.2 (-8.9, 6.4) | 0.753 |
| 3-month | 44.0 (35.5, 52.4) | 41.1 (35.3, 46.9) | 2.9 (-7.7, 13.5) | 0.593 |
| 6-month | 48.7 (40.9, 56.5) | 40.2 (34.6, 45.9) | 8.4 (-1.1, 17.9) | 0.082 |
| 12-month | 48.6 (40.1, 57.0) | 45.5 (39.4, 51.6) | 3.1 (-7.0, 13.2) | 0.546 |
| **Emotional functioning (EF)** |  |  |  |  |
| Baseline | 56.4 (51.7, 61.1) | 56.5 (52.5, 60.5) | -0.1 (-6.3, 6.0) | 0.969 |
| 3-month | 66.4 (62.2, 70.5) | 60.2 (55.2, 65.2) | 6.2 (-0.2, 12.6) | 0.059 |
| 6-month | 63.2 (58.0, 68.5) | 57.7 (52.9, 62.4) | 5.6 (-1.5, 12.6) | 0.123 |
| 12-month | 63.7 (55.7, 71.7) | 60.0 (54.6, 65.5) | 3.7 (-6.1, 13.4) | 0.451 |
| **Cognitive functioning (CF)** |  |  |  |  |
| Baseline | 77.4 (72.8, 82.1) | 71.8 (66.0, 77.6) | 5.6 (-1.80, 13.1) | 0.137 |
| 3-month | 74.3 (69.8, 78.8) | 71.7 (66.9, 76.5) | 2.6 (-4.1, 9.4) | 0.443 |
| 6-month | 80.7 (76.2, 85.1) | 68.9 (64.3, 73.4) | 11.8 (5.4, 18.2) | <0.001 |
| 12-month | 76.9 (70.4, 83.4) | 72.9 (67.9, 77.9) | 4.0 (-4.6, 12.6) | 0.355 |
| **Social functioning (SF)** |  |  |  |  |
| Baseline | 50.5 (42.9, 58.0) | 47.5 (42.4, 52.5) | 3.0 (-6.1, 12.1) | 0.518 |
| 3-month | 53.1 (47.1, 59.2) | 48.5 (42.5, 54.5) | 4.6 (-4.2, 13.5) | 0.302 |
| 6-month | 58.4 (50.9, 66.0) | 50.0 (43.4, 56.6) | 8.4 (-1.2, 18.1) | 0.088 |
| 12-month | 57.5 (50.3, 65.0) | 60.0 (52.9, 67.1) | -2.5 (-12.5, 7.5) | 0.620 |

**Table S4** Change of functional scales in the EORTC QLQ-C30

The pooled mean and 95%CI were estimated based on the linear mixed models with multiple imputation (10 imputed data sets).
Abbreviations: EORTC QLQ-C30, European Organisation for Research and Treatment of Cancer Quality of Life Questionnaire Core 30; QoL, Quality of Life.

| **Symptom/Item scales of the EORTC QLQ-C30** | **Intervention group (IG)**  **Mean (95%CI)** | **Control group (CG)**  **Mean (95%CI)** | **Mean difference (IG – CG)**  **(95% CI)** | **p-value** |
| --- | --- | --- | --- | --- |
| **Fatigue (FA)** |  |  |  |  |
| Baseline | 58.8 (53.2, 64.5) | 59.3 (54.5, 64.1) | -0.5 (-7.9, 7.0) | 0.906 |
| 3-month | 52.5 (45.8, 59.2) | 55.5 (50.6, 60.3) | -3.0 (-11.2, 5.3) | 0.481 |
| 6-month | 47.1 (39.5, 54.6) | 60.0 (55.1, 64.8) | -12.9 (-21.7, -4.0) | 0.004 |
| 12-month | 51.0 (42.8, 59.2) | 52.3 (46.0, 58.5) | -1.2 (-12.3, 9.8) | 0.823 |
| **Nausea and vomiting (NV)** |  |  |  |  |
| Baseline | 15.3 (11.0, 19.7) | 19.5 (15.7, 23.4) | -4.2 (-10.1, 1.7) | 0.160 |
| 3-month | 14.0 (9.7, 18.4) | 18.6 (13.4, 23.7) | -4.5 (-11.2, 2.1) | 0.181 |
| 6-month | 9.1 (5.7, 12.4) | 17.2 (11.4, 23.0) | -8.1 (-14.8, -1.4) | 0.018 |
| 12-month | 10.8 (4.3, 17.3) | 11.7 (6.5, 16.9) | -0.9 (-9.4, 7.6) | 0.828 |
| **Pain (PA)** |  |  |  |  |
| Baseline | 36.3 (30.0, 42.6) | 38.0 (31.8, 44.1) | -1.7 (-10.5, 7.1) | 0.706 |
| 3-month | 33.8 (27.7, 39.9) | 33.3 (27.7, 38.9) | 0.5 (-8.3, 9.2) | 0.914 |
| 6-month | 31.9 (25.1, 38.6) | 32.6 (26.8, 38.5) | -0.8 (-9.5, 7.9) | 0.863 |
| 12-month | 32.9 (25.3, 40.4) | 35.9 (29.3, 42.6) | -3.1 (-12.8, 6.7) | 0.537 |
| **Dyspnoea (DY)** |  |  |  |  |
| Baseline | 41.2 (33.9, 48.5) | 40.8 (34.7, 46.8) | 0.4 (-9.1, 9.9) | 0.930 |
| 3-month | 36.1 (29.3, 42.8) | 43.9 (38.0, 49.7) | -7.8 (-16.6, 1.1) | 0.086 |
| 6-month | 34.8 (27.6, 42.0) | 45.5 (39.7, 51.3) | -10.7 (-19.7, -1.7) | 0.020 |
| 12-month | 33.5 (24.5, 42.4) | 39.1 (32.5, 45.6) | -5.6 (-17.3, 6.1) | 0.345 |
| **Insomnia (SL)** |  |  |  |  |
| Baseline | 39.6 (32.9, 46.4) | 45.7 (39.7, 51.7) | -6.1 (-15.1, 3.0) | 0.188 |
| 3-month | 38.8 (32.1, 45.5) | 41.8 (35.2, 48.4) | -3.0 (-12.5, 6.5) | 0.531 |
| 6-month | 35.3 (28.1, 42.6) | 47.6 (40.6, 54.6) | -12.3 (-22.3, -2.3) | 0.016 |
| 12-month | 37.2 (27.2, 47.2) | 45.0 (37.4, 52.6) | -7.8 (-21.0, 5.3) | 0.233 |
| **Appetite loss (AP)** |  |  |  |  |
| Baseline | 37.9 (30.7, 45.0) | 41.9 (35.6, 48.1) | -4.0 (-13.5, 5.5) | 0.410 |
| 3-month | 28.7 (22.8, 34.5) | 38.8 (32.7, 44.9) | -10.2 (-18.6, -1.7) | 0.018 |
| 6-month | 23.7 (17.2, 30.3) | 35.4 (28.4, 42.4) | -11.6 (-21.5, -1.8) | 0.021 |
| 12-month | 25.7 (16.5, 34.9) | 26.5 (17.6, 35.3) | -0.7 (-14.6, 13.1) | 0.914 |
| **Constipation (CO)** |  |  |  |  |
| Baseline | 17.6 (12.4, 22.7) | 23.1 (17.7, 28.5) | -5.5 (-12.9, 1.9) | 0.146 |
| 3-month | 12.8 (8.4, 17.1) | 19.6 (12.8, 26.5) | -6.9 (-14.8, 1.01) | 0.087 |
| 6-month | 13.6 (8.8, 18.4) | 18.5 (12.8, 24.1) | -4.9 (-12.2, 2.5) | 0.194 |
| 12-month | 12.3 (6.2, 18.4) | 14.0 (9.0, 19.0) | -1.7 (-9.3, 6.0) | 0.671 |

**Table S5** Change of symptom and item scales in the EORTC QLQ-C30
The pooled mean and 95%CI were estimated based on the models with multiple imputation (10 imputed data sets).
Abbreviations: EORTC QLQ-C30, European Organisation for Research and Treatment of Cancer Quality of Life Questionnaire Core 30; QoL, Quality of Life.

Table 5 continued

| **Symptom/Item scales of the EORTC QLQ-C30** | **Intervention group (IG)**  **Mean (95%CI)** | **Control group (CG)**  **Mean (95%CI)** | **Mean difference (IG – CG)**  **(95% CI)** | **p-value** |
| --- | --- | --- | --- | --- |
| **Diarrhoea (DI)** |  |  |  |  |
| Baseline | 20.6 (14.1, 27.1) | 21.8 (17.4, 26.4) | -1.3 (-9.3, 6.6) | 0.739 |
| 3-month | 15.6 (10.2, 20.9) | 17.6 (13.2, 22.1) | -2.1 (-8.9, 4.7) | 0.547 |
| 6-month | 14.7 (9.0, 20.3) | 15.3 (11.0, 19.6) | -0.6 (-7.9, 6.6) | 0.866 |
| 12-month | 14.9 (6.8, 22.9) | 18.1 (11.4, 24.8) | -3.3 (-11.7, 5.2) | 0.448 |
| **Financial difficulties (FI)** |  |  |  |  |
| Baseline | 17.0 (11.2, 22.8) | 18.4 (14.3, 22.6) | -1.4 (-8.6, 5.7) | 0.695 |
| 3-month | 25.5 (16.2, 34.7) | 27.0 (21.4, 32.7) | -1.6 (-13.1, 9.9) | 0.789 |
| 6-month | 23.5 (14.2, 32.7) | 26.7 (21.1, 32.2) | -3.2 (-14.7, 8.3) | 0.583 |
| 12-month | 25.4 (14.2, 36.7) | 24.8 (17.5, 32.1) | 0.7 (-13.3, 14.6) | 0.925 |

**Table S5** Change of symptom and item scales in the EORTC QLQ-C30.
Note: The pooled mean and 95%CI were estimated based on the linear mixed models with multiple imputation (10 imputed data sets).
Abbreviations: EORTC QLQ-C30, European Organisation for Research and Treatment of Cancer Quality of Life Questionnaire Core 30; QoL, Quality of Life.

|  | **Intervention group (IG)**  **Mean (95%CI)** | **Control group (CG)**  **Mean (95%CI)** | **Mean difference (IG – CG)**  **(95% CI)** | **p-value** |
| --- | --- | --- | --- | --- |
| **IPQ-R (Coherence)** |  |  |  |  |
| Baseline | 16.26 (15.41, 17.10) | 16.30 (15.70, 16.91) | -0.05 (-1.06, 0.97) | 0.930 |
| 3-month | 16.97 (16.22, 17.71) | 16.65 (15.88, 17.42) | 0.31 (-0.79, 1.42) | 0.579 |
| 6-month | 16.45 (15.41, 17.49) | 16.63 (15.68, 17.58) | -0.18 (-1.67, 1.30) | 0.808 |
| 12-month | 16.93 (15.69, 18.17) | 16.71 (15.79, 17.62) | 0.23 (-1.50, 1.96) | 0.790 |
| **PRA-D** |  |  |  |  |
| Baseline | 29.68 (28.54, 30.81) | 29.45 (27.81, 31.09) | 0.23 (-1.73, 2.18) | 0.820 |
| 3-month | 30.11 (28.97, 31.25) | 29.26 (28.19, 30.34) | 0.85 (-0.65, 2.34) | 0.266 |
| 6-month | 29.70 (28.32, 31.09) | 29.09 (27.54, 30.64) | 0.61 (-1.54, 2.76) | 0.574 |
| 12-month | 29.18 (27.56, 30.80) | 29.42 (28.18, 30.67) | -0.24 (-2.24, 1.76) | 0.811 |
| **API-DM** |  |  |  |  |
| **Preference for participation** |  |  |  |  |
| Baseline | 51.33 (48.66, 54.00) | 52.83 (50.59, 55.06) | -1.49 (-4.91, 1.93) | 0.392 |
| 3-month | 50.28 (47.76, 52.81) | 53.87 (51.28, 56.46) | -3.59 (-7.27, 0.09) | 0.056 |
| 6-month | 51.20 (47.82, 54.58) | 55.81 (52.97, 58.64) | -4.60 (-9.25, 0.04) | 0.052 |
| 12-month | 50.12 (47.10, 53.14) | 53.92 (50.72, 57.13) | -3.80 (-8.76, 1.16) | 0.128 |
| **Preference for information** |  |  |  |  |
| Baseline | 96.04 (95.02, 97.07) | 96.48 (95.50, 97.46) | -0.43 (-1.81, 0.95) | 0.539 |
| 3-month | 96.72 (95.69, 97.76) | 94.39 (92.87, 95.91) | 2.33 (0.52, 4.15) | 0.012 |
| 6-month | 96.27 (94.90, 97.65) | 95.42 (94.14, 96.70) | 0.85 (-1.19, 2.89) | 0.407 |
| 12-month | 95.66 (93.54, 97.78) | 94.26 (92.25, 96.28) | 1.39 (-1.75, 4.54) | 0.368 |
| **DCS** |  |  |  |  |
| Baseline | 22.48 (18.30, 26.67) | 22.13 (18.55, 25.72) | 0.35 (-5.11, 5.82) | 0.900 |
| 3-month | 24.83 (19.81, 29.85) | 23.15 (19.14, 27.17) | 1.68 (-4.76, 8.11) | 0.608 |
| 6-month | 21.62 (17.07, 26.16) | 27.17 (22.02, 32.33) | -5.56 (-12.29, 1.18) | 0.105 |
| 12-month | 20.33 (14.08, 26.59) | 24.48 (20.20, 28.75) | -4.14 (-11.29, 3.00) | 0.249 |
| **HLS-EU-Q6** |  |  |  |  |
| Baseline | 2.76 (2.63, 2.89) | 2.73 (2.60, 2.85) | 0.03 (-0.14, 0.21) | 0.701 |
| 3-month | 2.67 (2.57, 2.78) | 2.69 (2.56, 2.82) | -0.01 (-0.19, 0.16) | 0.871 |
| 6-month | 2.76 (2.62, 2.91) | 2.63 (2.51, 2.75) | 0.13 (-0.02, 0.29) | 0.093 |
| 12-month | 2.76 (2.58, 2.93) | 2.67 (2.50, 2.83) | 0.09 (-0.17, 0.35) | 0.481 |

**Table S6** Change of scores in the secondary PROs outcomes
Note: IPQ-R=Illness Perception Questionnaire, PRA-D=Patient Reaction Assessment, API-DM=German modified version of the Autonomy Preference Index,
DCS=Decisional Conflict Scale, HLS-EU-Q6=European Health Literacy Survey

The pooled mean and 95%CI were estimated based on the linear mixed models with multiple imputation (10 imputed data sets).

**References:**

1. Bodner TE. What Improves with Increased Missing Data Imputations? Struct Equ Modeling. 2008;15(4):651-75. https://doi.org/10.1080/10705510802339072

2. Rubin DB. Multiple imputation for nonresponse in surveys. John Wiley and Sons. 1987:XXIX+258 pp.

3. Fiero MH, Hsu C-H, Bell ML. A pattern-mixture model approach for handling missing continuous outcome data in longitudinal cluster randomized trials. Stat Med. 2017;36(26):4094-105. https://doi.org/10.1002/sim.7418

4. Leurent B, Gomes M, Faria R, Morris S, Grieve R, Carpenter JR. Sensitivity Analysis for Not-at- Random Missing Data in Trial-Based Cost-Effectiveness Analysis: A Tutorial. Pharmacoeconomics. 2018;36(8):889-901. https://doi.org/10.1007/s40273-018-0650-5

5. Billingham LJ, Abrams KR. Simultaneous analysis of quality of life and survival data. Stat Methods Med Res. 2002;11(1):25-48. https://doi.org/10.1191/0962280202sm269ra
